# On the Singular Perturbations for Fractional Differential Equation

**DOI:** 10.1155/2014/752371

**Published:** 2014-02-09

**Authors:** Abdon Atangana

**Affiliations:** Institute for Groundwater Studies, Faculty of Natural and Agricultural Sciences, University of the Free State, Bloemfontein 9301, South Africa

## Abstract

The goal of this paper is to examine the possible extension of the singular perturbation differential equation to the concept of fractional order derivative. To achieve this, we presented a review of the concept of fractional calculus. We make use of the Laplace transform operator to derive exact solution of singular perturbation fractional linear differential equations. We make use of the methodology of three analytical methods to present exact and approximate solution of the singular perturbation fractional, nonlinear, nonhomogeneous differential equation. These methods are including the regular perturbation method, the new development of the variational iteration method, and the homotopy decomposition method.

## 1. Introduction

Singular perturbation problems have constantly been playing an outstanding character in cooperation in the theory of differential equations and in their applications to the physical world. With a loose knot speaking, singularly perturbed differential equations are pigeonholed by the presence of quite a lot of essentially different scales. The existence of these scales gives one a small parameter and thus permits one to use perturbation methods. It is, however, typical of singular perturbation problems that a straight forward perturbation fails to be uniformly valid. Traditionally, this type of behaviour, which is abundant in applications, has been tackled by a large variety of methods, such as matched asymptotic expansions and averaging. Nevertheless, many fundamental issues still remain unresolved. Singular perturbation theory is a rich and ongoing area of exploration for mathematicians, physicists, and other researchers. The methods used to tackle problems in this field are many. The more basic of these include the method of matched asymptotic expansions [1–4] and WKB approximation for spatial problems [5–7], and in time, the Poincaré-Lindstedt method [8, 9], the method of multiple scales [10, 11], and periodic averaging [12, 13].

While searching in the literature, it is observed that attention has not been paid to the extension of this class of ordinary and partial differential equations to the concept of fractional calculus. In order to accommodate readers that are not acquainted with the concept of fractional calculus, it is perhaps important to recall that fractional calculus, the art of noninteger order integrals and derivatives, has gained an interesting momentum in recent years [14–20]. The applications are ranging from pure and applied mathematics. It is easy to find experts working in this field because of its beauty, while others look for applications. The main aim of this work is to provide impetus, motivation, and to bring together researchers and scientists working in the fields of fractional calculus and perturbation method by providing some useful analytical techniques to derive exact and approximate solutions of this class of equations. The general singular perturbation fractional differential equation considered in this paper is given as follows:

(1) Dtαu(t)+εAu(t)+Bu(t)=fε(t),u(a)=g(ε),  u(b)=h(ε),

where *t* ∈ [*a*, *b*], *A*, and *B* are linear and nonlinear operators, respectively, *D*_*t*_^*α*^ is the fractional order derivative, *f* is known function, and *ε* is a small parameter. We will present the review of the fractional calculus in the next section.

## 2. Review of Fractional Calculus

A significant tip is that the fractional derivative at a point *x* is a local property only when *a* is an integer; in noninteger cases we cannot say that the fractional derivative at *x* of a function *f* depends only on values of *f* very near *x*, in the way that integer-power derivatives unquestionably do. For that reason, it is anticipated that the philosophy involves some class of boundary conditions, involving information on the function further out. To use a metaphor, the fractional derivative requires some tangentialvision. As far as the existence of such a philosophy is concerned, the brass tacks of the subject were laid by Liouville in a paper from 1832.

### 2.1. Definitions

**Definition 1 (Riemann-Liouville integral)**

The classical form of fractional calculus is given by the Riemann-Liouville integral. The theory for periodic functions therefore including the “boundary condition” of repeating after a period is the Weyl integral. The Riemann-Liouville integral of order *α* of a function *f*(*x*) is given by

(2) Da  t−αf(x)=Ia  tαf(x)=1Γ(α)∫ax(x−t)α−1f(t)dt.

**Definition 2 (Hadamard fractional integral)**

The Hadamard fractional integral is introduced by Hadamard [21] and is given by the following formula:

(3) Da  t−αf(x)=1Γ(α)∫ax(log⁡(t2))α−1f(t)tdt.

**Definition 3 (Riemann-Liouville fractional derivative)**

The corresponding derivative is calculated using Lagrange's rule for differential operators. Computing *n*th order derivative over the integral of order (*n* − *α*), the *α* order derivative is obtained. It is important to remark that *n* is the nearest integer bigger than *α*. Not in the same line of ideas like in the case of classical Newtonian derivatives, a fractional derivative is defined via a fractional integral as follows.

(4) Da  tαf(x)=dn[Ia  tnf(x)]dxn=1Γ(n−α)dndxn∫ax(x−t)α−1f(t)dt.

**Definition 4 (Caputo fractional derivative)**

There is another alternative for computing fractional derivatives that is the Caputo fractional derivative. It was introduced by Caputo and Michel in his 1967 paper [22]. In contrast to the Riemann-Liouville fractional derivative, when solving differential equations using Caputo's definition, it is not necessary to define the fractional order initial conditions. Caputo's definition is illustrated as follows:

(5) D0Ctαf(x) =1Γ(n−α)∫ax(x−t)α−1dndtnf(t)dt.

The above definitions are commonly used in pure and applied mathematics.

**Definition 5**

If we make use of the primary definition of an integral as being the antiderivative, it would be perhaps tempting to propose another definition of fractional derivative, which would consist of replacing *α* by −*α* in right hand of (2) as follows:

(6) Da  tαf(x)=1Γ(α)∫ax(x−t)−α−1f(t)dt.

The above expression is much simpler to handle on one hand; on the other hand, it is representing only with a single integral. The function in this case does not need to be differentiable in order to compute its fractional derivative. Note that the properties of this derivative will not be presented in this present work.

**Definition 6**

The Laplace transform is a widely used integral transform with many applications in physics and engineering. The Laplace transform of the function *f* is defined as follows:

(7) ℒ(f(x))(s)=∫0∞e−sxf(x)dx.

### 2.2. Properties of Fractional Calculus

Two properties of the Laplace transform can be used to define the fractional integral operator as follows:

(8) ℒ(I(f))=ℒ(∫0xf(τ)dτ)(s)=1sℒ(f(x))(s),ℒ(I2(f))=ℒ(∫0x∫0x1f(τ)dτ dx1)(s)=1s2ℒ(f(x))(s).

Now using the induction method, we arrive at the following:

(9) ℒ(In(f))=1snℒ(f(x))(s).

From the above equation, we can obtain the following:

(10) ℒ(In(f))=1snℒ(f(x))(s)⟹In(f)=ℒ−1(1snℒ(f(x))(s)).

It is well-known from the convolution theorem of the Laplace transform that

(11) ℒ(f∗g(x))(s)=ℒ(f(x))(s)·ℒ(g(x))(s).

Now from the previous formula, if we chose for *g*(*x*) = *x* ^*α*−1^, with the information of (10), the fractional integral operator can be defined as follows:

(12) Da  tαf(x)=1Γ(α)ℒ−1(ℒ(f(x))(s)·ℒ(g(x))(s)),Datαf(x)=1Γ(α)(f∗g(x))=1Γ(α)∫ax(x−t)α−1f(t)dt.

According to the classical Newtonian derivative, the Leibniz rule is given as

(13) dn[f·g]dxn=∑k=0n(nk)f(k)(x)gn−k(x).

Not in the same line of idea like in the case of classical Newtonian derivatives, the Leibniz rule for the fractional derivative is given as follows

(14) Da  tα(f(x)g(x)) =∑k=0n(pk)g(k)(x)Da  tp−kf(x)  −1n!Γ(−p)∫0x(x−t)−p−1f(t)dt  ×∫txgn+1(ψ)(t−ψ)ndψ.

The above result is achieved using a simple integration by part. Now if in addition, the functions *f* and *g* are continuous together with their entire derivative on [0, *x*], then

(15) lim⁡n→∞⁡1n!Γ(−p)∫0x(x−t)−p−1f(t)dt  ×∫txgn+1(ψ)(t−ψ)ndψ=0.

In that case, we have the following Leibniz rule for fractional derivative:

(16) Da  tα(f(x)g(x))=∑k=0n(pk)g(k)(x)Da  tp−kf(x).

Let us observe that Laplace transform of the fractional derivative with both Riemann-Liouville and Caputo as follows:

(17) ℒ(D0Ctαf(x))(s)=sαF(s)−∑k=0n−1sα−k−1f(k)(0),(n−1<α≤n).

The above use the usual initial conditions or values of the functions. On the other hand, we have the Riemann-Liouville such that

(18) ℒ(D0  tαf(x))(s)=sαF(s)−∑k=0n−1sα−k−1Da  tp−k−1f(0),(n−1≤α<n).

The above makes use of the unusual initial value or conditions of the function, therefore it is not suitable for the real world problems.

## 3. Analytical Methods

In this section, make use of some existing analytical methods to present the exact and approximate solution of some singular perturbation fractional equation (1). It is very important to note that, if the nonlinear term in (1) is zero, then it is straightforward to derive the exact solution of (1) using the Laplace transforms method as follows.

### 3.1. Laplace Transform Method

The first step in this method consists of applying the Laplace transform on both sites of equation to obtain for the case of Caputo derivative as follows

(19) sαU(s)−∑k=0n−1sα−k−1u(k)(0)+εA(U(s))=Fε(s).

For simplicity, we assume that the linear part of the transformation can be written as follows:

(20) A(U(s))=U(s)∑k=0n−1gk(s,ε)−h(s,ε).

We can factorize the term with *U*(*s*, *ε*); then solving *U*(*s*, *ε*), we obtain

(21) U(s,ε)=Fε(s)+∑k=0n−1sα−k−1u(k)(0)+h(s,ε)∑k=0n−1gk(s,ε)+sα.

Under the condition that the right hand side of the above equation is Laplace invertible; then, we obtain the exact solution of (1) as follows:

(22) u(x,ε)=ℒ−1(Fε(s)+∑k=0n−1sα−k−1u(k)(0)+h(s,ε)∑k=0n−1gk(s,ε)+sα).

In case that the nonlinear part is different to zero, other analytical methods in this area have been proposed.

### 3.2. The Regular Perturbation Method

There are several existing perturbation methods, for instance, match asymptotic expansion, WKB method, the multiple-scale analysis, and regular perturbation methods [23–26]. The regular perturbation method is perhaps the most naive way to try to solve problem (1) and this consists of assuming an expansion of the Poincare type as follows:

(23) u(x,ε)=∑n=0∞un(x)εn.

On substituting the above in problem (1), one obtains a sequence as follows:

(24) P0:  M(u0,0)=0,P1:  M1(u0,u1,0)=0,Pn:  Mn(u0,u1,u2,…,un)=0.

The above method has some limitations, for instance, the nonlinear part can be very difficult to handle for strong linearity like *u* ^*n*^, *n* ≥ 3, also the *M*_*n*_(*u*_0_, *u*_1_, *u*_2_,…, *u*_*n*_) = 0 can also be very difficult to solve. Both regular and singular perturbation theory are frequently used in physics and engineering. Perturbation theory can fail when the system can transition to a different phase of matter, with a qualitatively different behavior, that cannot be modeled by the physical formulas put into the perturbation theory, for instance, a solid crystal melting into a liquid. In some cases, this failure manifests itself by divergent behavior of the perturbation series. Such divergent series can sometimes be resumed using techniques such as Borel resummation. However, perturbation techniques can be also used to find approximate solutions to nonlinear differential equations. Examples of techniques used to find approximate solutions to these types of problems are the Lindstedt-Poincaré technique, the method of harmonic balancing, and the method of multiple time scales.

### 3.3. Some Iteration Methods

Many iterations methods have been proposed in the literature so far. Some of them provide powerful tools to handle nonlinear and linear equations ranging from ordinary derivatives to fractional derivatives. These techniques are, namely, the homotopy perturbation methods (HPM) [27], the homotopy decomposition method [28–30], the Adomian decomposition method [31, 32], the variational iteration method [33, 34], and the Bernstein collateral method [35]. These mentioned methods have been used to handle many problems across the sciences. In some cases, the above iterations produce the exact solutions and also approximate solutions for strong nonlinear equations.

## 4. Applications

This section is devoted to the discussion underpinning the application of Laplace and iteration method for solving singular linear and nonlinear perturbation fractional differential equation.

### 4.1. Application of Laplace Transform: Linear Case

**Example 7**

We will consider the following singular perturbation fractional differential equation:

(25) D0Ctαu(t)+εdu(t)dt+td2u(t)dt2+du(t)dt=2ε(e−t2ε2(−1+ε(−1+2t2ε)))π−t1−απ ×(Hyper  geometric  PFQ   ×((12,1),(1−α2,32−α2,1−t2ε2))).

To solve the above equation, we apply the Laplace transform on both sides of (25) to obtain

(26) sαU(s)−sα−1u(0)+(ε+1)(sU(s)−u(0))+ℒ(td2u(t)dt2)=Fε(s).

Here, the term is

(27) ℒ(td2u(t)dt2)=−dds[s2U(s)−su(0)−u′(0)].

Now substituting the above expression (27) into (26), we obtain

(28) sαU(s)−sα−1u(0)+(ε+1)(sU(s)−u(0))+2sU(s)+s2U′(s)=Fε(s).

The above equation can be reformulated as follows:

(29) j(s,ε,α)U(s)+U′(s)=G(s,ε,α).

The general solution of the above equation is given as

(30) U(s,ε)=∫0sexp⁡[∫0x′j(x,ε,α)dxG(x′,ε,α)dx′+c]exp⁡[∫0sj(x,ε,α)dx].

Now to find the exact solution of (25), we apply the inverse Laplace transform on both sides of (30) to obtain

(31) erf⁡[xε] =ℒ−1(U(s,ε)) =ℒ−1(∫0sexp⁡[∫0x′j(x,ε,α)dxG(x′,ε,α)dx′+c]exp⁡[∫0sj(x,ε,α)dx]).

The above is the exact solution of (25).

**Example 8**

Let us consider the following singular perturbation fractional differential equation:

(32) D0Ctαu(t)+tdu(t)dt+εd2u(t)dt2+du(t)dt=12etεεα+(1+t)εcosh⁡⁡[tε] +(e−tε((−ε)α(αΓ(−α)+Γ(1−α,−tε))−e2tεεαΓ[1−α,tε])×(2Γ(1−α))−1)+ε3sinh⁡⁡[tε].

To solve the above equation, we apply the Laplace transform on both sides to obtain

(33) sαU(s)−sα−1u(0)+(sU(s)−u(0))−(U(s)−sU′(s))+ε(s2U(s)−su(0)−u′(0))=Fε(s).

Again after rearranging, we put the above equation in the following form.

The above equation can be reformulated as follows:

(34) V(s,ε,α)U(s)+U′(s)=L(s,ε,α).

The general solution of the above equation is given as

(35) U(s,ε)=∫0sexp⁡[∫0x′V(x,ε,α)dxL(x′,ε,α)dx′+c]exp⁡[∫0sV(x,ε,α)dx].

Now to find the exact solution of (32), we apply the inverse Laplace transform on both sides of (35) to obtain

(36) sinh⁡⁡[εx] =ℒ−1(U(s,ε)) =ℒ−1(∫0sexp⁡[∫0x′V(x,ε,α)dxL(x′,ε,α)dx′+c]exp⁡[∫0sV(x,ε,α)dx]).

The above is the exact solution of (32).

### 4.2. Iterations and Regular Perturbation Methods: Nonlinear Case

In this section, we compare the methodology of some iteration methods, namely, homotopy decomposition method, variational iteration method, and the regular perturbation method, for solving the nonlinear singular perturbation fractional differential equation. The basic idea of HDM can be found in [28–30] and the variational iteration method can be found in [33, 34].

**Example 9**

Let us consider the following nonlinear singular perturbation fractional ordinary differential equation:

(37) D0Ctαu(t)+εd2u(t)dt2+du(t)dt+u2(t) =et+ε(2+et+ε+ϵ−Γ(1−α,t)Γ(1−α)),u(0)=exp⁡⁡(ϵ).

#### 4.2.1. Solution via the HDM

To solve (37) via homotopy decomposition method, we first apply on both sides of the inverse operator of _0_^*C*^ *D*_*t*_^*α*^ to obtain

(38) u(t)=u(0) +1Γ(α)∫0t(t−x)1−α ×(−εd2u(x)dt2−du(x)dt−u2(x) +ex+ε(2+ex+ε+ϵ−Γ(1−α,x)Γ(1−α))).

The next step is to assume that the solution of (38) is in a series form as

(39) u(t)=lim⁡p→1⁡u(t,p)=lim⁡p→1⁡∑n=0∞pnun(t).

Now substituting (39) into (38), we obtain

(40) ∑n=0∞pnun(t) =u(0)+pΓ(α)∫0t(t−x)1−α×(−εd2∑n=0∞pnun(x)dt2−d∑n=0∞pnun(x)dt−(∑n=0∞pnun(x))2+ex+ε(2+ex+ε+ϵ−Γ(1−α,x)Γ(1−α))).

After comparing the term of same power of *p*, we obtain

(41) u0(t)=u(0),u1(t)=∫0t(t−x)1−α×(−εd2u0(x)dx2−du0(x)dx−(u0)2+ex+ε×(2+ex+ε+ϵ−Γ(1−α,x)Γ(1−α)))dx,u2(t)=∫0t(t−x)1−α×(−εd2u0(x)dx2−du0(x)dx−(2u1u0))dx.

In general, for all *n* ≥ 3, we have the following iteration formula:

(42) un(t) =∫0t(t−x)1−α(−εd2un−1(x)dx2−dun−1(x)dx−(∑i=0n−1uiun−1−i))dx.

After integration, we obtain the following solutions:

(43) u0(t)=exp⁡⁡(ε),u1(t)=exp⁡⁡(ε)t,u2(t)=exp⁡⁡(ε)t22,u3(t)=exp⁡⁡(ε)t36,un(t)=exp⁡⁡(ε)tnn!,u(t)=lim⁡n→∞⁡∑k=0nexp⁡⁡(ε)tnn!=exp⁡⁡[x+ε].

This is the exact solution of (37).

#### 4.2.2. Variational Iteration Method

In its initial development, the essential nature of the method was to construct the following correction functional for general equation when *α* is a natural number:

(44) un+1(t) =un(t)+∫0tλ(t,τ)[−∂mu(τ)∂τm+A(wn(τ))+B(wn(τ))+f(τ)]dτ,

where *λ*(*t*, *τ*) is the so-called Lagrange multiplier [33] and *w*_*n*_(*x*, *t*) is the *n*-approximate solution. Therefore, in their work, they apply the new development of the VIM proposed in [33] to find the Lagrange multiplier. In this new VIM, the first step of the basic character of the method is to apply the Laplace transform on both sides of (1) to obtain

(45) sαU(s)−∑k=0n−1sα−k−1u(k)(0)=ℒ[−εA(u(t))−B(u(t))+fε(t)].

The recursive formula of (1) can now be used to put forward the main recursive method connecting the Lagrange multiplier as

(46) un+1(s)=un(s) +λ(s)[sαU(s)−∑k=0n−1sα−k−1u(k)(0) −ℒ[εA(un(t))+B(un(t))−fε(t)]].

Now considering that is *ℒ*[−*εA*(*w*_*n*_(*x*, *t*)) − *B*(*w*_*n*_(*x*, *t*)) + *f*_*ε*_(*t*)] the restricted term, the Lagrange multiplier can be obtained as [35]

(47) λ(s)=−1sα.

Now applying the inverse Laplace transform on both sides of (46), we obtain the following iteration:

(48) un+1(t)=un(t) −ℒ−1[1sα[sαun(s)−∑k=0n−1sα−k−1u(k)(0)−ℒ[εA(un(t))+B(un(t))−fε(t)]]].

Rearranging, we arrive at the following iteration formula:

(49) un+1(t) =ℒ−1[[−1sαℒ[εA(un(t))+B(un(t))−fε(t)]]],ℒ−1{∑k=0n−1sα−k−1u(k)(0)}=u0(t).

Therefore, in the case of (37), we have

(50) u0(t)=exp⁡⁡(ε),u1(t)=−e2εtαΓ(1+α)+2et+ε(Γ(α)−Γ(α,t))Γ(α) +et+εε(Γ(α)−Γ(α,t))Γ(α) +2−αe2t+2ε(Γ(α)−Γ(α,t))Γ(α) −∫0tes+ϵ(t−s)−1+α(Γ(α)−Γ(α,s))Γ(α)Γ(1−α)ds.

In this case, with this method, the component will be very difficult to obtain.

#### 4.2.3. Regular Perturbation Method

To solve (37) with regular perturbation technique, we assume that the solution is in series form as

(51) u(t,ε)=∑n=0∞εnun(t).

Substituting the above in (37), we obtain

(52) D0Ctα(∑n=0∞εnun(t))+εd2dt2(∑n=0∞εnun(t))   +ddt(∑n=0∞εnun(t))+(∑n=0∞εnun(t))2  =et+ε(2+et+ε+ϵ−Γ(1−α,t)Γ(1−α)).

To be consistent, we put in the Taylor series all functions of *ε* in the right hand side of (52), as follows:

(53) et+ε(2+et+ε+ϵ−Γ(1−α,t)Γ(1−α)) =(2et+etΓ(1−α,t)Γ(1−α))  ×∑n=0∞(ε)nn!+e2t∑n=0∞(2ε)nn!+et∑n=0∞(ε)n+1n!.

Now substituting (53) into (52) we obtain

(54) D0Ctα(∑n=0∞εnun(t))+εd2dt2(∑n=0∞εnun(t))   +ddt(∑n=0∞εnun(t))+(∑n=0∞εnun(t))2  =(2et+etΓ(1−α,t)Γ(1−α))    ×∑n=0∞(ε)nn!+e2t∑n=0∞(2ε)nn!+et∑n=0∞(ε)n+1n!.

Comparing the term of the same terms of *ε*, we arrive at the following fractional differential equations:

(55) ε0:  D0Ctα(u0(t))+ddt(u0(t))+u02  =2et+etΓ(1−α,t)Γ(1−α)+e2t,ε1:  D0Ctα(u1(t))+ddt(u1(t))+d2dt2(u0)+2u0u1   =3et+etΓ(1−α,t)Γ(1−α)+e2t,ε2:  D0Ctα(u2(t))+ddt(u2(t))+d2dt2(u1)   +u0u2+u12  =3et+etΓ(1−α,t)Γ(1−α)+e2t,ε3:  D0Ctα(u3(t))+ddt(u3(t))+d2dt2(u2)   +2u0u3+2u1u2  =3et+etΓ(1−α,t)Γ(1−α)+e2t,ε4:  D0Ctα(u4(t))+ddt(u4(t))+d2dt2(u3)+u22+2u0u4   =3et+etΓ(1−α,t)Γ(1−α)+e2t,ε5:  D0Ctα(u5(t))+ddt(u5(t))   +d2dt2(u4)+2u0u5   +2u2u3+2u1u4  =3et+etΓ(1−α,t)Γ(1−α)+e2t,ε6:  D0Ctα(u6(t))+ddt(u6(t))   +d2dt2(u5)+2u1u5+u32   +2u2u4+2u0u6  =3et+etΓ(1−α,t)Γ(1−α)+e2t.

The next concern in this approach is to solve for *u*_0_(*t*) and substitute it in *ε* ^1^ to find *ε* ^1^ and so on. To solve for *u*_0_(*t*), we can employ the methodology of homotopy decomposition method as follows. First, we transform the equation to fractional integral equation as

(56) u0(t)=u0(0)+1Γ(α)   ×∫0t(t−x)1−α(ddτ(u0(τ))+u02−2eτ−eτΓ(1−α,τ)Γ(1−α)−e2τ)dτ.

After we assume that the solution can be in series form as discussed earlier, we obtain the following recursive formula:

(57) u00(t)=u0(0),u01(t)=1Γ(α)∫0t(t−x)1−α×(ddτ(u00(τ))+u002−2eτ−eτΓ(1−α,τ)Γ(1−α)−e2τ)dτ,u02(t)=1Γ(α)∫0t(t−x)1−α×(ddτ(u01(τ))+2u01(τ)u00(τ))dτ,u0n(t)=1Γ(α)∫0t(t−x)1−α×(ddτ(u0n−1(τ))+∑i=0n−1u0iu0n−1−i)dτ.

Then, the solution of *ε* ^0^ will be

(58) u0(t)=∑k=0∞u0k(t).  

With the above in hand, we can substitute it into equation *ε* ^1^ and use the same procedure to find *u*_1_(*t*) in the form of

(59) u1(t)=∑k=0∞u1k(t).

We continue this process until we obtain the desired number of term of the series solution as

(60) un(t)=∑k=0∞unk(t).

Then, the solution of (37) via the regular perturbation method will be

(61) u(t,ε)=∑n=0∞εnun(t).

**Remark 10**

The regular perturbation method required a lot of time and also the method is very difficult to handle.

## 5. Conclusion

We make use of the Laplace transform and some analytical techniques to derive exact and approximate solutions to both nonlinear and linear nonhomogeneous singular perturbation fractional ordinary differential equations. We proposed a relatively new fractional derivative using both the Riemann-Liouville fractional integral and the basic definition of the integral as being the antiderivative. We presented some difficulties encountered while using the regular perturbation method. We demonstrate that the iteration method can be successfully used to solve singular perturbation fractional differential equations.
